# An Upper Bound on the Power of DNA to Distinguish Pedigree Relationships

**DOI:** 10.3390/genes16050492

**Published:** 2025-04-26

**Authors:** Maarten Kruijver

**Affiliations:** Institute of Environmental Science and Research, Auckland 1142, New Zealand; maarten.kruijver@esr.cri.nz

**Keywords:** identity by descent, kinship, relationship testing, investigative genetic genealogy

## Abstract

**Background/Objectives:** Dense genetic marker panels are increasingly used in kinship analysis for the identification of distant relatives. As more markers are available, it is possible to pinpoint IBD segments more precisely and more reliably, ultimately approaching close to continuously observed IBD. This study investigates the evidential value obtained for discrimination between common pedigree relationships if IBD is observed continuously across the autosomal genome without error. In the continuous case, the evidential value is limited only by the pedigree relationship and the recombination rates. **Methods:** We conducted simulations to generate IBD segments across the autosomal genome for individuals with defined pedigree relationships. The evidential value for relationship discrimination was then calculated exactly from the underlying model, assuming no genotyping error and full genome coverage. **Results:** The simulations show that the ability to distinguish pedigree relationships quickly diminishes as relationships become more distant. First cousins can be distinguished from second cousins with 99.9% accuracy which drops to 94% when distinguishing second and third cousins. Relationships with the same expected degree of relatedness can be discriminated using continuously observed IBD, although the effectiveness decreases with more distant relationships. **Conclusions:** Continuous IBD observation establishes a theoretical upper bound on the power to distinguish relationships if a large but finite number of markers is used. The findings provide a benchmark for evaluating kinship analyses based on finite genetic marker panels.

## 1. Introduction

The use of DNA to estimate family relationships is commonplace in areas ranging from animal conservation and crop improvement to paternity testing and missing persons identification [1]. There is a long history of using independent genetic markers and comparing the likelihood of the observations under two alternative hypotheses, dating at least back to Essen-Möller [2,3]. More recently, the use of dense SNP marker panels and direct-to-consumer genetic testing [4,5] has enabled the identification of more distant relatives, broadening the scope of applications to large-scale genetic genealogy. On a smaller scale, these methods have also been adopted in criminal investigations [6,7].

The move from a small number of genetic markers usually residing on different chromosomes to dense panels that contain many markers on the same chromosome has required changes to the way the genetic data are interpreted statistically [8,9,10]. Markers on the same chromosome can be inherited together, which complicates the calculation of pedigree likelihoods [11]. At a more fundamental level, there is increased awareness that the level of relatedness is not just described by the pedigree but can be seen as varying continuously throughout the genome if viewed as the distance to their most recent common genetic ancestor at that location [12]. Although a pedigree-based notion of relatedness is the most widely used one in everyday life, it is not always the most useful definition in other contexts. For example, in medical studies realised relatedness may be more relevant than pedigree-based relatedness when considering genetic factors that influence disease risk or treatment response [13]. That notwithstanding, the current work adopts the pedigree perspective motivated by the relevance to human identification. Specifically, we ask the question: *how well can the different pedigree relationships be distinguished using genetic data from two persons*? To shed light on this question, we consider an idealised setting in which it is possible to observe identity by descent (IBD) continuously along the autosomal genome for a pair of persons. In practice, it is only possible to observe identity by state (IBS) for discrete genetic data, and this can be further complicated by genotyping errors. Because a segment can only be IBD if it is IBS, we may view relationship testing based on genetic marker data as an inferential problem with incomplete information, where the full information is IBD observed for a continuous genome. As a consequence, the power of discrimination of a procedure based on continuously observed IBD serves as an upper bound on what could be achieved in practice.

To answer the central question of this work, we simulate large numbers of samples of continuously observed IBD for various pedigree relationships. IBD is observed for two persons and these persons are assumed to not be related otherwise than through the pedigree. Based on the simulated data, we estimate how informative the data are for distinguishing relationships (e.g., first versus second cousins) by studying the empirical likelihood ratio distributions [14] and related summary statistics. These likelihood ratios are calculated using an exact likelihood model for the IBD segments. Both the simulations and calculations make use of the **ibdsegments** package for **R** [15]. The code that generates the results presented in the article is available online (https://github.com/mkruijver/an_upper_bound_paper_data accessed on 31 March 2025).

The power to distinguish pedigree relationships is assessed in several ways. When comparing two specific relationships, denoted as $H_{1}$ and $H_{2}$, the (log) likelihood ratio distributions for $H_{1}$ true and $H_{2}$ true show how well the relationships can be distinguished based on the data. If the curves have little overlap, then there is strong discriminative power. Numerical summaries are also given. Rates of misleading evidence, $\mathrm{Pr}(\mathrm{LR}>1|H_{2})$ and $\mathrm{Pr}(\mathrm{LR}<1|H_{1})$, inform us how often the evidence from the data is misleading rather than informative. The median LR’s for $H_{1}$ true and $H_{2}$ true inform us how strongly the data typically support the correct hypothesis. If the rate of misleading evidence is small and the median LR of the $H_{1}$ true distribution is large, it is possible to reliably distinguish $H_{1}$ and $H_{2}$. Further, we estimate the accuracy of a classifier that assigns $H_{1}$ when LR≥1 and $H_{2}$ otherwise. If $H_{1}$ and $H_{2}$ are equally probable, then the accuracy is the average of one minus the rates of misleading evidence. The accuracy is a single number that summarises how well relationships can be distinguished. Besides the power to distinguish pairs of relationships, we also investigate the power to identify the correct relationship from a set of more than two relationships.

Many previous studies have investigated the power of DNA to distinguish pedigree relationships if a certain set of markers or specific methodology is used. For example, Tillmar and Kling recently compared the performance of several SNP kits when used with different statistical methods [10]. In contrast, we directly study the information content of the IBD distribution, establishing an upper bound on the potential performance of any method. As far as we are aware, this is the first attempt to directly apply an exact likelihood-based approach to observations of continuous IBD segments. The modelling approach and assumptions are, however, fairly standard. Specifically, we adopt Haldane’s model for recombination [16] which assumes that crossovers occur according to a Poisson process along the genetic map, and we use sex-averaged recombination rates to simplify calculations. This approach has been widely used in variants of the Lander–Green algorithm [17,18]. The application to modelling continuous IBD goes back to Donnelly [19], who provided results about the probability of sharing some DNA for different pedigree relationships, effectively envisioning today’s investigative genetic genealogy [20]. The current work does not only consider the probability of sharing some DNA but extends to a complete model for IBD segment observations.

## 2. Materials and Methods

### 2.1. Pedigree Relationships and IBD

We discuss several pairwise pedigree relationships (e.g., half-siblings, first cousins, etc.). Following the notation of [21], P denotes a pedigree, and a pairwise relationship between pedigree members *a* and *b* is a triple (a,b,P). At any point on the genome, each pedigree founder can be assigned two founder haplotype labels representing two homologous chromosomes. Two pedigree members are said to be IBD1 at a location on a chromosome if they inherited the same founder haplotype. If the pedigree members share two founder haplotypes, then we say they are IBD2 (or double IBD), and if they do not share anything, then they are IBD0.

**Figure 1:**
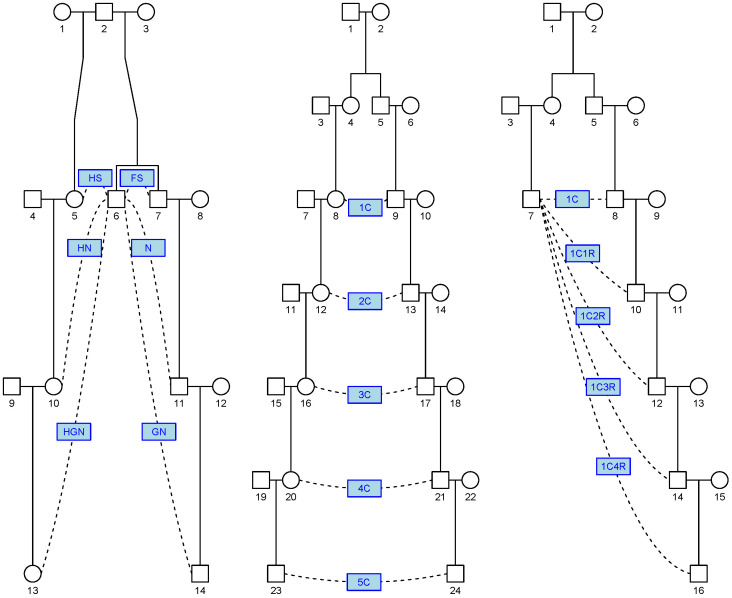
Common pedigree relationships. The left-hand side shows half-siblings (abbr. HS) (5, 6) and full siblings (abbr. FS) (6, 7). Descendants of the full siblings are the nephew (abbr. N) (6, 11) and great-nephew (abbr. GN) (6, 14). The corresponding half-relationships are HN (6, 10) and HGN (6, 13). The middle pedigree illustrates cousin relationships 1C (first cousins) up to 5C (fifth cousins), i.e., pairs of linear descendants of the full siblings (4, 5). The right-hand side illustrates cousin relationships with removal, e.g., 1C1R stands for first cousins once removed.

Several pedigree relationships were studied. Figure 1 illustrates some relationships and their abbreviations. In particular, we refer to the following relationships:

- Linear relationships: These are relationships between a person and their parent, grandparent (abbreviated GP), great-grandparent (abbreviated GGP), and so on. Beyond the GGP, these relationships are abbreviated as $G^{n}$GP with n=2,3,…. The GP relationship is referred to as a linear relationship of degree 2, so $G^{n}$GP is a linear relationship of degree n+2.
- Cousin relationships: These are relationships between linear descendants of two full siblings. First cousins are labelled 1C, second cousins 2C, and so on. Removal is indicated by the number and the letter R such that first cousins once removed becomes 1C1R. The full sibling relationship could be considered 0th cousins but this relationship is excluded from this study to simplify the analysis by considering the IBD status to be binary (0 or 1).
- Avuncular relationships: These are relationships between a person and (descendants of) a child of their full sibling. The uncle–nephew relationship is abbreviated as N. Great-nephew is abbreviated as GN. For n≥2, this relationship is abbreviated as $G^{n}$N.
- Half-cousin relationships: These are linear descendants of two half-siblings. These relationships are prefixed with the letter H, for example, H1C2R stands for half first cousins twice removed.

The ability to distinguish pedigree relationships based on genetic marker data relies on IBD [22]. For a given pedigree relationship, the expected proportion of a chromosome that is IBD can be computed. For example, full siblings are expected to share approximately 50% of their autosomal genome IBD on average: about 25% as IBD2 (both alleles identical by descent), 50% as IBD1 (one allele shared), and 25% as IBD0 (no alleles identical by descent). The realised quantities are different as a consequence of Mendelian inheritance and recombination [23]. For example, it is possible that full siblings are not IBD on one chromosome, while on another chromosome, they are partly IBD1 and partly IBD2.

### 2.2. Relatedness Inference

In a recent review, Kling et al. discuss various approaches to infer genetic relationships between two or more persons [7]. The authors broadly distinguish two categories of approaches. The exploratory approaches aim to estimate one or more summary statistics from genetic data that are informative of the degree of relatedness or relationship. This category covers a wide range of approaches ranging from counting the number of shared alleles at STR loci [24] or estimating the kinship coefficient using dense SNP data (e.g., [25,26]) to directly identifying IBD segments using dense [27] or relatively sparse marker sets [28] and considering their length and/or count [29].

Kling et al. also discuss likelihood ratio approaches where the probability of the observed data under one hypothesis is compared to the probability of the observed data under another hypothesis. Likelihood ratio approaches are widely used in paternity testing [30] and other kinship testing using small panels of mostly independent markers [31]; however, these approaches are still emerging for use with denser marker panels for which the population genetic modelling is more complex [32].

Likelihood ratio approaches can make use of all available data to quantify the evidence for one hypothesis versus a second one [14]. The summary statistics produced in exploratory approaches are generally not sufficient to convey all the information that is informative of the data-generating hypothesis. This means that efficiency may be lost when these exploratory approaches are applied to discriminate between two pedigree relationships instead of a likelihood ratio-based approach that models all the data. In practice, likelihood ratio approaches are sensitive to model misspecification and measurement error, for instance when population genetic assumptions are not met or measurements are unreliable, which may cause increased rates of misleading evidence [33,34]. A practical benefit of exploratory approaches is that these may be more amenable to the use of statistics that can be estimated robustly, for instance when genotyping error or population genetics are complicating factors. The distinction between exploratory approaches and likelihood ratio approaches is blurred by score-based likelihood ratio approaches in which likelihood ratios are assigned based on summary statistics, or scores, instead of the raw data.

### 2.3. Continuous IBD

We briefly sketch how computations and simulations involving continuous IBD are implemented in the **ibdsegments** package. Further details are available in the package documentation and the references therein. We applied a likelihood ratio approach where the data are continuously observed IBD segments without error. We viewed the chromosome as a continuum and assumed Haldane’s model of recombination [16] with equal recombination rates for males and females. For a given pedigree relationship, the IBD state at a point on a continuous chromosome of length *L* centiMorgan (cM) is a random variable $\left(X_{t},t\geq 0\right)$ taking values in a finite set *S* of identity states for 0≤t≤L. In the remainder, we keep the exposition simple and in line with investigative genetic genealogy practice by considering only the two identity states IBD0 and IBD1: S={0,1}. The algorithms and software used support other choices for the set *S* of identity states as well. For example, one could also chose the three states IBD0 to IBD2, the nine condensed, or the fifteen detailed identity states [35]. The current work was restricted to IBD0 and IBD1, so the IBD state could be conveniently viewed as an indicator variable, which simplified the methodology. Because the full sibling relationship had an IBD2 probability greater than zero, it was not considered.

#### 2.3.1. IBD Vector and the Hidden Markov Model

**Figure 2:**
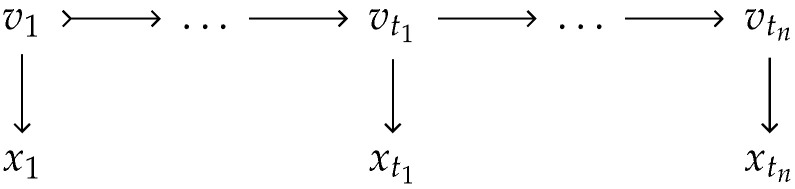
Hidden Markov Model relating the continuously observed IBD state ($X_{t}$) to the underlying hidden pedigree IBD state (*V*).

Applying Haldane’s model for recombination, the IBD process can be seen as a continuous-time Hidden Markov Model [36], where the observed variable is $X_{t}$. The hidden component is the binary IBD vector $V_{t}$ that tracks for each meiosis whether the grandpaternal or grandmaternal haplotype segment is passed down. See Figure 2 for a schematic illustration of the relationship between $X_{t}$ and $V_{t}$. This setup is also the basis for the widely used Lander–Green algorithm [17].

For a pedigree with $n_{nf}$ non-founders, there are $2\times n_{nf}$ meioses tracked by the IBD vector, so $V_{t}$ has length $2\times n_{nf}$. Because each element of the vector is binary (0 or 1), there are $2^{2\times n_{nf}}=:n_{v}$ possible IBD vectors. Thus, the state space of the Hidden Markov Model has $n_{v}$ elements and can be identified with the integers $0,\ldots ,n_{v}-1$. Note that in practice, the size of the state space can be reduced by exploiting symmetries [37,38] which further complicates implementation details. Crossovers for individual meioses have inter-arrival times with λ=0.01 crossovers per cM. It follows that the transition intensity $q_{ab}$ from state *a* to state *b* of the whole IBD vector with $a,b\in 0,\ldots ,n_{v}-1$ is:

$$q_{ab}=\left\{\begin{array}{cc} -\sum_{c\neq a}q_{ac}, & \mathrm{if}\;a=b,\\ 0.01, & \mathrm{if}\;\mathrm{Hamming}(a,b)=1,\\ 0, & \mathrm{otherwise}, \end{array}\right.$$

where Hamming(a,b) denotes the Hamming distance between the binary representations of integer states *a* and *b* (i.e., the number of bits by which they differ). The transition intensity $q_{ab}$ represents the instantaneous rate of transitioning from IBD state *a* to state *b*. The rate $q_{ab}=0.01$ applies only when the binary representation of *a* and *b* differ by exactly one bit, i.e., differ by one crossover in the pedigree. The diagonal entries $q_{aa}$ are chosen so that each row of the intensity matrix sums to zero as is required for a continuous-time Markov process.

#### 2.3.2. Simulating Continuous IBD

For all but the simplest pedigree relationships, the IBD process $\left(X_{t}\right)$ cannot be easily analysed using standard probability distributions. Although individual meioses yield exponential inter-arrival times for crossovers, the joint effect of crossovers throughout the pedigree leads to non-standard IBD segment length distributions. However, it is possible to simulate large numbers of realisations of $X_{t}$ at little computational cost by simulating the individual crossovers represented in $V_{t}$ and determining $X_{t}$ from $V_{t}$. The **ibdsegments** package was used to simulate Mendelian inheritance and crossover for the 22 autosomes. The simulation procedure worked by randomly flipping bits of the unobserved IBD vector with exponential inter-arrival times. A sex-averaged genetic map [39] was used with Haldane’s model for recombination events [16]. For example, Table 1 shows data obtained from the result of a simulation for a pair of first cousins at a chromosome of length 100 cM. There are 4 non-founders in the pedigree, so there are 8 meioses and $2^{8}=256$ possible IBD vectors. Each row of **(a)** shows a segment for which the IBD state does not change. The underlying IBD vector ($V_{t})$ is shown in **(b)**. Note that while the table shows a realisation of the simulation for a single chromosome, the simulations in this study comprised 22 chromosomes of different lengths.

#### 2.3.3. Expectation and Variance of Total IBD

For many relationships, the single point probability distribution of $\left(X_{t}\right)$—also referred to as identity coefficients—is reproduced in textbooks, and software tools are available to compute it for any relationship [40]. We denote the three identity coefficients for IBD0, IBD1, and IBD2 as $\kappa_{0}$, $\kappa_{1}$, and $\kappa_{2}$, that is $\kappa_{1}=\mathrm{Pr}\left(\mathrm{IBD}1\right)$. In the **ibdsegments** package, these identity coefficients can be computed for arbitrary pairs of pedigree members. The package uses a brute-force algorithm that exhaustively iterates through all possible, equally probable IBD vectors. For each vector, founder haplotype labels are dropped through the pedigree to determine if the identity state is realised and the probability has to be incremented.

In the current work, we were also interested in the full stochastic process $\left(X_{t},t\geq 0\right)$ observed continuously over t∈[0,L] with $X_{t}$ taking values 0 and 1 corresponding to the IBD0 and IBD1 states. For a chromosome of length *L*, we defined total IBD, *T*, as the combined length of all IBD1 segments on the chromosome:

(1) $$T=\int_{0}^{L}I_{\left\{X_{t}=1\right\}}dt,$$

where $I_{A}$ denotes the indicator function that is 1 for *A* and 0 otherwise. The expectation and variance of *T* can be relatively easily computed. Since $\mathrm{Pr}(X_{t}=1)$ does not depend on *t*, we may write the expected value of *T* as the product of $\kappa_{1}$ and *L*:

(2) $$E\left(T\right)=\int_{0}^{L}\kappa_{1}dt=\kappa_{1}L.$$

The variance of *T* can be calculated as $\mathrm{Var}\left(T\right)=E\left(T^{2}\right)-E{\left(T\right)}^{2}$, where the term $E(T^{2})$ depends on the joint distribution $X_{u},X_{v}$ for 0≤u,v≤L. In Haldane’s model of recombination, this distribution depends only on the distance |u−v|, so we may compute the variance of *T* by double numerical integration of the two-locus IBD probability [41,42,43].

(3) $$E\left(T^{2}\right)=\int_{0}^{L}\int_{0}^{L}I_{\left\{X_{u}=X_{v}=1\right\}}dvdu=\int_{0}^{L}\int_{0}^{L}\kappa_{11}(|u-v|)dvdu,$$

where $\kappa_{11}(|u-v|)$ denotes the two-locus IBD probability for loci separated by |u−v| cM.

When more than one chromosome is considered, we denote the total IBD across the chromosomes as $T_{+}$. The chromosomes are assumed to be independent so expectations and variances can be summed.

#### 2.3.4. Probability of No IBD: Pr(T=0)

Computing the probability of no IBD across a chromosome, Pr(T=0), requires more elaborate calculations involving the IBD process. Pr(T=0) can be written as

(4) $$\mathrm{Pr}\left(T=0\right)=\mathrm{Pr}(X_{t}=0,\forall t\in \left[0,L\right]).$$

The IBD vector has to only take on values for which the observed IBD state is 0; however, neither the state nor the number of state transmissions (recombination events) are observed. The probability Pr(T=0) can be computed by conditioning on the number of recombination events in the pedigree—Poisson-distributed—and then enumerating all possible paths that are compatible with no IBD:

(5) $$\begin{array}{cc} \mathrm{Pr}(X_{t}=0,\forall t\in \left[0,L\right]) & =\sum_{k=0}^{\infty }\mathrm{Pr}\left(N=k\right)\mathrm{Pr}\left(X_{t}=0,\forall t\in \left[0,L\right]|N=k\right) \end{array}$$

(6) $$\begin{array}{cc} \; & \;\;\;\;\;=\sum_{k=0}^{\infty }\mathrm{Pr}\left(N=k\right)\sum_{v_{0},\ldots ,v_{k}\in V^{0}}p_{v_{0}}\prod_{j=1}^{k}p_{v_{j-1},v_{j}}, \end{array}$$

where

- $\mathrm{Pr}\left(N=k\right)=\exp\left(-\lambda \right)\lambda^{k}/k!$ since the number of recombination events in the pedigree is Poisson-distributed, N∼Poisson(λ), with $\lambda =0.01\times L\times 2n_{nf}$, where $n_{nf}$ is the number of non-founders in the pedigree.
- The sum over *k* can be truncated after a finite value $k_{\max}$. Choosing the 1−ϵ-quantile of the Poisson distribution ensures the truncation error is smaller than ϵ.
- $V^{0}$ is the set of paths for which the IBD state remains 0.
- $p_{v_{0}}$ denotes the prior probability $\mathrm{Pr}(V_{0}=v_{0})$ of starting in $v_{0}$. This probability equals $1/n_{v}$ where $n_{v}$ is the number of IBD vectors.
- $p_{v_{j-1},v_{j}}$ is shorthand for $\mathrm{Pr}(V_{j}=v_{j}|V_{j-1}=v_{j-1})$.

In practice, explicitly enumerating all possible paths that are compatible with no IBD and summing over those is not feasible. Instead, the forward algorithm [44] is used to efficiently compute the probability of the observed sequence under the Hidden Markov Model. Although the IBD vector at each position is unobserved, only the states in $V^{0}$ are considered in the computation. The forward algorithm recursively computes, for each discrete step *m*, the probability of being in each compatible hidden state given the observations up to time *m*. Let $\alpha_{m}\left(v\right)=\mathrm{Pr}\left(V_{m}=v,\;\mathrm{all}\;\mathrm{observed}\;\mathrm{IBD}\;\mathrm{states}\;\mathrm{up}\;\mathrm{to}\;m\;\mathrm{are}\;0\right)$ for $v\in V^{0}$. Then, the forward recursion is given by:

$$\alpha_{0}\left(v\right)=1/n_{v},\;\mathrm{for}\;v\in V^{0},$$

$$\alpha_{m}\left(v\right)=\sum_{u\in V^{0}}\alpha_{m-1}\left(u\right)p_{u,v},\;\mathrm{for}\;v\in V^{0},$$

where $p_{u,v}=\mathrm{Pr}\left(V_{t}=v|V_{t-1}=u\right)$ is the transition probability from *u* to *v*. This transition probability is $1/n_{nf}$ if *u* and *v* differ by one bit and zero otherwise. The recursion proceeds up to $k_{\max}$. Finally, the probability of observing no IBD across the entire chromosome is obtained by summing over the forward probabilities:

(7) $$\mathrm{Pr}\left(X_{t}=0,\forall t\in \left[0,L\right]\right)=\sum_{k=0}^{k_{\max}}\mathrm{Pr}\left(N=k\right)\sum_{v\in V^{0}}\alpha_{k}\left(v\right).$$

#### 2.3.5. Likelihoods for IBD Segments

Computing the likelihood of a realisation of the continuous IBD process (such as displayed in Table 1) is approached similarly. As in the previous section, the key idea is to use the forward algorithm to efficiently sum over all unobserved IBD vectors that are compatible with the observed IBD segments. Let $t_{1},\ldots ,t_{n}$ be the endpoints of the *n* segments, and let $x_{i}$ denote the IBD status of the segment ending at $t_{i}$. The likelihood of the segment observations can be computed iteratively for each segment where the IBD vector is captured in the probability of $V_{t}$ at the start of the segment. Writing $v_{i-}$ for the unobserved state at the start of segment *i*,

(8) $$\begin{array}{cc} \mathrm{Pr}(x,t) & =\mathrm{Pr}(x_{1},t_{1},\ldots ,x_{n},t_{n}|H) \end{array}$$

(9) $$\begin{array}{cc} \; & \;\;\;\;\;\;\;\;\;\;\;\;\;=\mathrm{Pr}\left(x_{1},t_{1}|H\right)\prod_{i=2}^{n}\mathrm{Pr}\left(x_{i},t_{i}|x_{1},\ldots ,x_{i-1},t_{1},\ldots ,t_{i-1},H\right) \end{array}$$

(10) $$\begin{array}{cc} \; & \;\;\;\;\;\;\;\;\;\;\;\;\;\;\;\;=\prod_{i=1}^{n}\sum_{v_{i-}\in S^{x_{i}}}\mathrm{Pr}\left(x_{i},t_{i}|v_{i-},H\right)\mathrm{Pr}\left(v_{i-}|x_{1},\ldots ,x_{i-1},t_{1},\ldots ,t_{i-1},H\right). \end{array}$$

The probabilities $\mathrm{Pr}(x_{i},t_{i}|v_{i-},H)$ are computed analogous to the method of the previous section by summing over the forward probabilities. The probabilities of $v_{i-}$ are also obtained from the forward pass. Finally, since chromosomes are assumed to be independent, the likelihood ratio for a whole genome observation is the product of likelihood ratios per chromosome.

#### 2.3.6. Likelihood Ratios for IBD Segments

After simulating many realisations of the continuous IBD process for all considered pedigree relationships, we may compute likelihood ratios for the simulated data where $H_{1}$ is one relationship and $H_{2}$ is another. This yields an empirical distribution of likelihood ratios for $H_{1}$ true and $H_{2}$ true. Kernel density estimates of the $\log_{10}$ likelihood ratios are shown in a plot, and numerical summaries are also provided.

We also considered the power to distinguish between more than two relationships. In this scenario, likelihood ratios are computed for hypothesis pairs of the form where $H_{1}$ is a relationship and $H_{2}$ is all other relationships in the set under consideration. For example, when considering the relationships GP, HS, and N, we may have the proposition pair:

(11) $$\begin{array}{cc} H_{1}: & \mathrm{HS}, \end{array}$$

(12) $$\begin{array}{cc} H_{2}: & \bar{\mathrm{HS}}=\mathrm{GP},N. \end{array}$$

Assuming equal prior probabilities for all alternatives, the likelihood ratio is then

(13) $$\mathrm{LR}=\frac{\mathrm{Pr}(E|H_{1})}{\mathrm{Pr}(E|H_{2})}=\frac{\mathrm{Pr}(E|H_{\mathrm{HS}})}{\mathrm{Pr}(E|H_{\bar{\mathrm{HS}}})}=\frac{\mathrm{Pr}(E|H_{\mathrm{HS}})}{1/2\times \mathrm{Pr}\left(E|H_{\mathrm{GP}}\right)+1/2\times \mathrm{Pr}\left(E|H_{N}\right)},$$

where *E* denotes the whole-genome continuous IBD segment observations.

### 2.4. Exploring IBD and Segment Count Distributions

As a first experiment towards answering the main question posed in this work, we proceeded by establishing an overview of which relationships could be distinguished reliably on the basis of summary statistics of IBD sharing. For a set of relationships, the following summary statistics were computed:

- $\kappa_{1}$: the probability of (single) IBD at any point.
- The expectation and standard deviation of $T_{+}$ (total IBD), the combined length (cM) of all IBD segments.
- The expectation and standard deviation of the segment count.
- $\mathrm{Pr}(T_{+}=0$): the probability of not sharing any autosomal DNA across the 22 chromosomes.
- The expectation and standard deviation of $N_{0}$, the number of chromosomes for which there is no IBD.

These statistics were exactly computed. Relationships with large differences in the summary statistics were relatively easily distinguished. However, these summary statistics did not contain all the information contained in the IBD process, and there were relationships that were distinguishable based on the full data but not based on $T_{+}$. To illustrate this, we simulated 100,000 realisations of continuous IBD for all pedigree relationships and plotted the empirical distributions of the following:

- Segment count: the number of IBD segments across the autosomal genome.
- $T_{+}$ (total IBD): the combined length (cM) of all IBD segments.

### 2.5. Empirical LRs for Distinguishing Relationships Using Continuous IBD

The main question of this work was directly investigated by studying likelihood ratio distributions for continuously observed IBD. The following scenarios were investigated. First, the power to distinguish relationships with different $\kappa_{1}$ was investigated for

- Linear relationships;
- Cousin-type relationships.

Secondly, the power to distinguish relationships with identical $\kappa_{1}$ was investigated for $\kappa_{1}=1/2,1/4,1/8$.

## 3. Results

### 3.1. Exploring Total IBD and Segment Count

The complete pattern of IBD sharing for a pedigree relationship is described by a complex stochastic process. Fortunately, a large part of the story can be understood by studying simple summary statistics. Table 2 shows $\kappa_{1}$, the moments of $T_{+}$, the moments of the segment count, $\mathrm{Pr}(T_{+}=0)$, and the moments of $N_{0}$ for various relationships. We made several high-level observations:

- Close relationships (larger $\kappa_{1}$) could be reliably distinguished from distant relationships (smaller $\kappa_{1}$) because the distributions of total IBD were well separated.
- As the relationships became more distant, the total expected IBD decreased while the standard deviation increased relative to the expected value. This means that the distributions of total IBD had more overlap as relationships became more distant.
- Relationships with identical $\kappa_{1}$ had the same expected total IBD. For example, relationships with $\kappa_{1}=1/2$ included GP, N, and HS, and these were all expected to share 1696 cM. However, their IBD distributions were not the same as evidenced by differences in the standard deviations. Thus, it may be possible to distinguish these relationships based on data beyond total IBD, such as segment count.
- The differences in IBD distributions between relationships with the same $\kappa_{1}$ decreased quickly as relationships became more distant. The standard deviation of total IBD, Pr(total IBD = 0) and the expected value and standard deviation of the number of chromosomes without IBD all showed a similar trend of convergence within the groups of relationships with the same $\kappa_{1}$. It was therefore not possible to reliably distinguish higher-order relationships with the same $\kappa_{1}$.
- Many cousin-type relationships such as 2C and 1C2R could not be distinguished because the IBD distributions were identical. Donnelly [19] considered cousin-type relationships of the type “*s*th cousins *t* times removed” where s≥1 and t≥0 and showed that the IBD distribution depended on 2s+t.
- For higher-order relationships, there was a substantial probability that no DNA was shared at all. For fifth cousins, there was about a 69% probability of not sharing DNA. For fourth cousins, this probability was about 30%.

**Figure 3:**
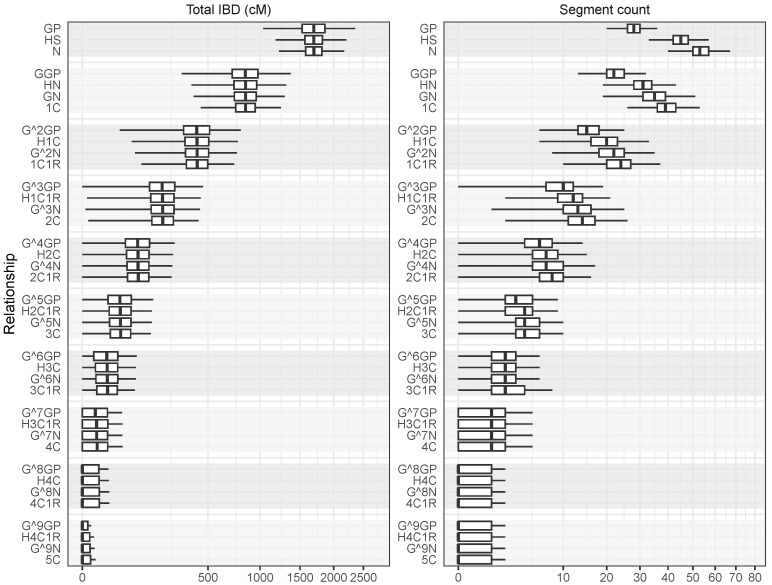
Realised total IBD (cM) and segment count for 100,000 simulations of pedigree relationships. Some relationships with the same expected degree of IBD such as N, GP, and HS can be distinguished based on segment count. As relationships become more distant, segment count quickly becomes less useful to distinguish between relationships with the same expected degree of IBD sharing.

Segment count is a useful statistic to distinguish relationships when the expected total IBD is identical. Figure 3 shows box plots of total IBD and segment count for 100,000 simulations of continuous IBD for the studied pedigree relationships. The figure demonstrates the extra resolution gained from considering the number of segments on top of total IBD. In particular, we observed the following:

- GP, HS, and N (all with $\kappa_{1}=1/2$) had mostly overlapping total IBD distributions but could be distinguished based on segment count.
- GGP, HN, GN, and 1C (all with $\kappa_{1}=1/4$) had mostly overlapping total IBD distributions and partly overlapping distributions of segment count.
- There appeared to be limited information in the segment count for distinguishing relationships with $\kappa_{1}$ smaller than, say, 1/4.
- As relationships become more distant, both the distributions of total IBD and segment count became highly skewed.

**Table 2:** IBD distributions summarised for common pedigree relationships.

Relationship	$\kappa_{1}$	Total IBD (cM)		Segment Count		$\mathrm{Pr}(T_{+}=0)$	$N_{T_{i}=0}$	
		Expected	S.d.	Expected	S.d.		Expected	S.d.
GP	1/2	1695.68	243.48	27.96	3.38	4.455 × 10^−22^	2.67	1.51
HS	1/2	1695.68	188.64	44.91	4.44	8.326 × 10^−37^	0.80	0.86
N	1/2	1695.68	174.35	53.39	4.94	2.091 × 10^−43^	0.49	0.68
GGP	1/4	847.84	196.31	22.46	4.02	2.264 × 10^−12^	6.80	2.13
HN	1/4	847.84	173.26	30.94	4.99	1.512 × 10^−15^	5.03	1.93
GN	1/4	847.84	167.35	35.17	5.56	1.828 × 10^−16^	4.61	1.87
1C	1/4	847.84	148.60	39.41	5.24	1.148 × 10^−21^	2.95	1.55
$G^{2}$GP	1/8	423.92	139.15	15.47	3.89	1.903 × 10^−7^	11.07	2.31
H1C	1/8	423.92	127.39	19.71	4.61	9.641 × 10^−9^	9.74	2.29
$G^{2}$N	1/8	423.92	124.22	21.83	5.05	3.854 × 10^−9^	9.37	2.27
1C1R	1/8	423.92	115.34	23.95	5.02	1.425 × 10^−10^	8.16	2.22
$G^{3}$GP	1/16	211.96	94.45	9.85	3.36	0.0001	14.66	2.18
H1C1R	1/16	211.96	87.97	11.97	3.86	2.860 × 10^−5^	13.79	2.24
$G^{3}$N	1/16	211.96	86.14	13.03	4.16	1.808 × 10^−5^	13.52	2.25
2C	1/16	211.96	81.46	14.09	4.19	4.761 × 10^−6^	12.76	2.28
$G^{4}$GP	1/32	105.98	63.05	5.99	2.72	0.0050	17.33	1.90
H2C	1/32	105.98	59.33	7.05	3.05	0.0025	16.80	1.97
$G^{4}$N	1/32	105.98	58.22	7.58	3.25	0.0019	16.62	2.00
2C1R	1/32	105.98	55.62	8.11	3.29	0.0011	16.18	2.05
$G^{5}$GP	1/64	52.99	41.86	3.52	2.12	0.0460	19.14	1.57
H2C1R	1/64	52.99	39.66	4.05	2.33	0.0322	18.84	1.64
$G^{5}$N	1/64	52.99	38.98	4.32	2.46	0.0283	18.73	1.66
3C	1/64	52.99	37.49	4.58	2.49	0.0211	18.49	1.71
$G^{6}$GP	1/128	26.49	27.80	2.03	1.61	0.1702	20.30	1.25
H3C	1/128	26.49	26.47	2.29	1.75	0.1415	20.14	1.30
$G^{6}$N	1/128	26.49	26.04	2.42	1.83	0.1321	20.07	1.32
3C1R	1/128	26.49	25.17	2.56	1.86	0.1144	19.94	1.36
$G^{7}$GP	1/256	13.25	18.51	1.15	1.21	0.3653	21.02	0.97
H3C1R	1/256	13.25	17.70	1.28	1.30	0.3319	20.93	1.01
$G^{7}$N	1/256	13.25	17.42	1.34	1.35	0.3200	20.89	1.02
4C	1/256	13.25	16.90	1.41	1.37	0.2979	20.82	1.05
$G^{8}$GP	1/512	6.62	12.37	0.64	0.89	0.5674	21.44	0.74
H4C	1/512	6.62	11.87	0.71	0.95	0.5400	21.39	0.77
$G^{8}$N	1/512	6.62	11.69	0.74	0.99	0.5296	21.37	0.78
4C1R	1/512	6.62	11.38	0.77	1.00	0.5110	21.34	0.80
$G^{9}$GP	1/1024	3.31	8.30	0.35	0.66	0.7293	21.69	0.56
H4C1R	1/1024	3.31	7.99	0.39	0.70	0.7109	21.66	0.58
$G^{9}$N	1/1024	3.31	7.87	0.40	0.72	0.7037	21.65	0.59
5C	1/1024	3.31	7.68	0.42	0.73	0.6912	21.63	0.60

**Figure 4:**
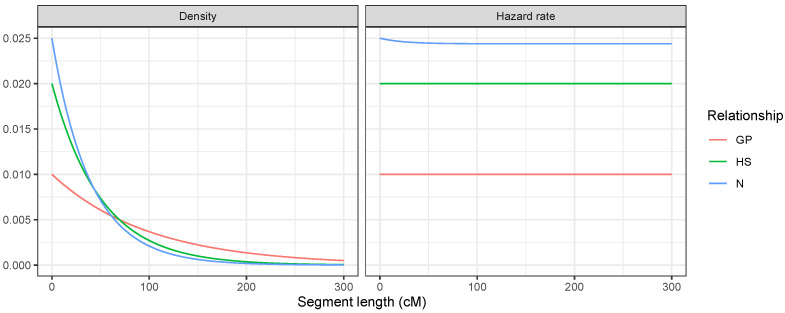
Segment length distributions at the start of a chromosome for three relationships with $\kappa_{1}=0.5$: grandparent–grandchild, half-siblings, and uncle–nephew.

As a final exploratory remark, we further illustrate differences between the IBD distributions of GP, HS, and N on a single chromosome. Assume that two persons are IBD at the start of a chromosome and consider the probability density function of the length of the segment, i.e., the distance until a recombination event disrupts the IBD. Figure 4 shows this probability density function (left) and the corresponding hazard rate [45,46] (right). For a continuous random variable with density f(x),x≥0 and distribution function F(x)=Pr(X≤x), the hazard rate is $h\left(x\right)=\frac{f(x)}{1-F(x)}=\frac{f(x)}{S(x)}$, where S(x)=1−F(x) is the survival function. The hazard rate represents the instantaneous rate at which two individuals switch from being IBD to not IBD, given that they were IBD up to that specific point on the chromosome. In our simulations, we observed the following:

- For HS, IBD occurred if and only if the shared parent passed down the same DNA to both offspring. Two meioses could break the IBD, so the segment length followed an Exp(0.02) distribution with a constant hazard rate of 0.02.
- For GP, only one meiosis broke the IBD, so the segment length followed an Exp(0.01) distribution with a constant hazard rate of 0.01.
- For N, there were two cases. Either the parent and uncle (who are siblings) were double IBD or single IBD. If they were double IBD, then it did not matter whether the grandpaternal or grandmaternal segment was transmitted from the parent to the nephew (this did not break the segment); however, two meioses broke the segment, yielding a hazard rate of 0.02. In the single IBD case, there were three meioses that broke the segment, and the hazard rate was 0.03. Both cases were equally probable at the start of the segment, so the hazard rate was 0.025. However, as the segment progressed, the double IBD could become a single IBD and vice versa without breaking the segment. Over longer segment lengths, the probability of being in the double IBD state increased, which slightly reduced the hazard rate.

### 3.2. Distinguishing Linear Relationships Using Continuous IBD

**Figure 5:**
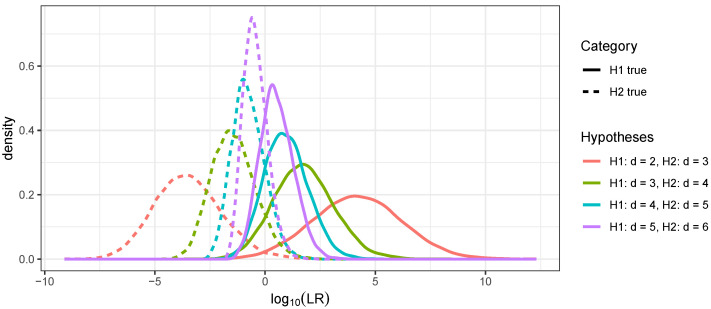
Kernel density estimates of 10,000 draws from LR distributions for distinguishing linear relationships of order *d* and d+1 for $H_{1}$ true (solid curves) and $H_{2}$ true (dashed curves).

First, likelihood ratio distributions are presented for distinguishing linear relationships of degree *d* and d+1 for d=2,…,5 based on continuous IBD. Note that d=2 is the grandparent relationship, and d=5 is the great-great-great-grandparent relationship. Figure 5 shows kernel density estimates of the log likelihood ratio distributions for $H_{1}$ true and $H_{2}$ true for d=2,…,5. As expected, the curves for $H_{1}$ true and $H_{2}$ true had more overlap as *d* increased, indicating that these relationships were less distinguishable. When distinguishing between grandparents and great-grandparents, likelihood ratios were often in the millions or more for $H_{1}$ true, although the range was substantial. Distinguishing great-grandparents (d=3) from great-great-grandparents (d=4) was harder, with likelihood ratios typically in the hundreds or thousands when $H_{1}$ was true (the median was just under 200). Further numerical summaries are presented in Table 3. Notably, the accuracy decreased from 98% when distinguishing second- and third-degree linear relationships to 77% when distinguishing linear relationships of fifth and sixth degree.

### 3.3. Distinguishing Cousin Relationships Using Continuous IBD

**Figure 6:**
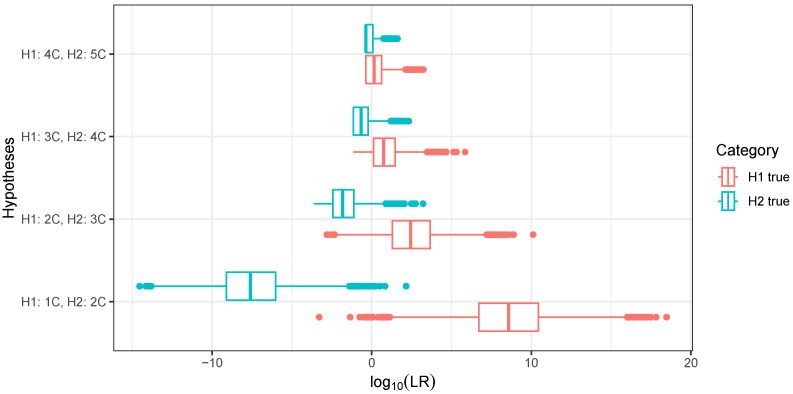
Boxplots of 10,000 draws from likelihood ratio distributions for distinguishing between *s*th and (s+1)th cousins for $H_{1}$ true and $H_{2}$ true.

Figure 6 shows boxplots of 10,000 draws from the LR distributions for distinguishing between cousin relationships of degree one to five. Boxplots were used instead of density estimates, because there were many samples with no IBD segments at all, especially for fifth cousins, which caused a point mass in the LR distribution. The boxplots show that first cousins could be distinguished from second cousins with LRs often in the billions in favour of the correct relationship. Distinguishing second cousins from third cousins was considerably harder with LRs typically in the hundreds or thousands. A numerical summary of the same data is presented in Table 4. The table shows that the rate of misleading evidence increased sharply for higher-degree cousins. There was about a 6% chance of misleading evidence when distinguishing second and third cousins. This increased to 30% when distinguishing fourth from fifth cousins.

### 3.4. Distinguishing Relationships with Identical $\kappa_{1}$

**Figure 7:**
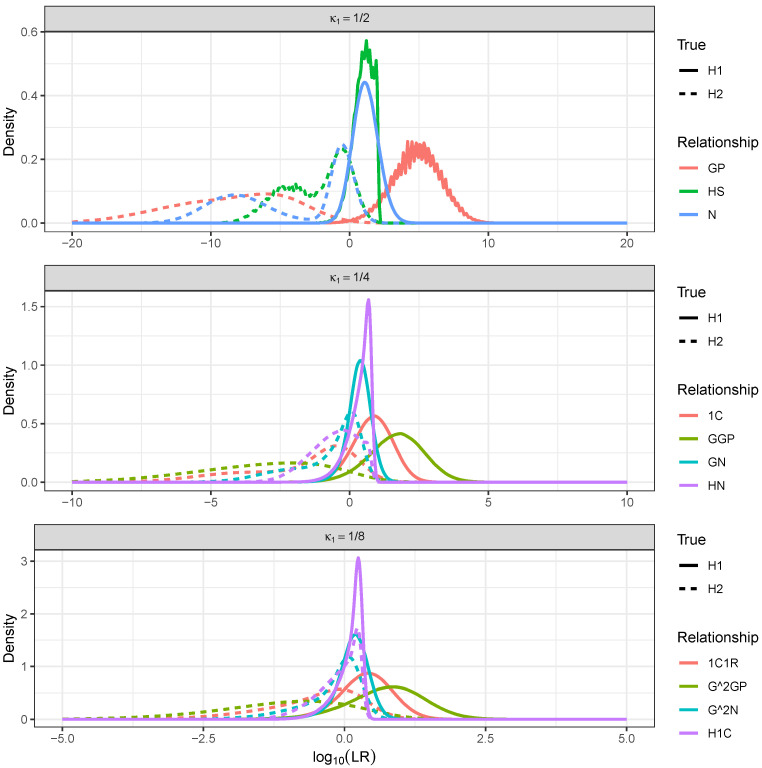
Density estimates of likelihood ratio distributions for distinguishing relationships with identical $\kappa_{1}$ for $H_{1}$ true and $H_{2}$ true. For each value of $\kappa_{1}$ and each relationship, likelihood ratios comparing $H_{1}$ (the relationship) and $H_{2}$ (another relationship with the same $\kappa_{1}$) were sampled (1,000,000 of each).

It is well known that the total IBD length is informative of the degree of the relationship. It is not clear how much information there is in the IBD segments besides their combined length, especially when it comes to distinguishing relationships with the same $\kappa_{1}$. Likelihood ratio distributions for distinguishing relationships with $\kappa_{1}=1/2$, $\kappa_{1}=1/4$, and $\kappa_{1}=1/8$ are presented in Figure 7. Numerical summaries are provided in Table 5. For each set of relationships with identical expected total IBD (identical $\kappa_{1}$), 100,000 random samples of the likelihood ratio distributions were taken for $H_{1}$ true and $H_{2}$ true. $H_{1}$ was a relationship (e.g., GP), and $H_{2}$ was the complement of this relationship within the set of relationships with the same $\kappa_{1}$. For example, for $H_{1}:\mathrm{GP}$, the complement would be $H_{2}:\bar{GP}=\left\{\mathrm{HS},N\right\}$. The alternatives in the composite hypothesis received equal weight in the likelihood of $H_{2}$.

Figure 7 and Table 5 demonstrate clearly that the power to discriminate relationships with the same $\kappa_{1}$ quickly diminished as the degree of the relationships increased. The accuracy decreased and the rates of misleading evidence increased with the degree of the relationships. Second-degree relationships ($\kappa_{1}=1/2$) could be distinguished especially well. The GP relationship was often distinguished from the others (HS and N) with likelihood ratios in the millions or more; the median was 4.8 on a $\log_{10}$ scale. There was less information in the IBD process for distinguishing HS and N. This was also apparent from Figure 3, which showed that the segment count distribution of HS and N had considerable overlap. Note that the $\log_{10}$ likelihood ratio curves looked jagged, especially for GP ($H_{1}$ true) and HS (both $H_{1}$ true and $H_{2}$ true). This was not an effect of the small sample size but was caused by point masses in the chromosome-wise likelihood ratio distributions. For the GP relationship, the expected number of chromosomes with no IBD was 2.67, and the standard deviation was 1.51 (see Table 2).

Third-degree relationships $\kappa_{1}=1/4$ were less distinguishable than first-degree relationships. Again, the linear relationship (GGP) was most distinguishable from the rest; however, the accuracy for this relationship dropped to 94%, down from over 99% for second-degree relationships. There was little power to discriminate fourth-degree relationships $\kappa_{1}=1/8$. The linear relationship ($G^{2}$GP) was most separate from the others and could be distinguished with an accuracy of only 84%. The median $\log_{10}$ LR for that relationship was less than one for the $H_{1}$ true samples.

## 4. Discussion

Our results demonstrated that the ability to distinguish pedigree relationships quickly diminished as relationships became more distant. For example, first cousins could be distinguished from second cousins with 99.9% accuracy, second from third with 94% accuracy, and this dropped to 81% when distinguishing third from fourth cousins. We demonstrated that there was useful information in the IBD distribution beyond the total length of shared segments. This additional information could be used to distinguish relationships with the same degree. This was effective for distinguishing second-degree relationship. Grandparents could be separated from half-siblings and nephews with over 99% accuracy. There was less power to distinguish half-siblings and nephews. As the degree of the relationships increased, there was less information in the shared segments that could be used to distinguish relationships of the same degree. Half-nephews and grand-nephews were distinguished from other third-degree relationships with only 75% accuracy. First cousins could be distinguished with 85% accuracy and great-grandparents with 94% accuracy. There was very little information to distinguish the different fourth-degree relationships.

The results of this study provide an upper bound on the power for distinguishing relationships if IBD is observed continuously and without error. The upper bound of what is achievable in practice using dense SNP panels is lower. As relationships become more distant, the IBD segments become shorter and increasingly hard to detect.

Several simplifying assumptions were made in this study. Notably, sex-averaged crossover rates were used to simplify the calculations. It is well known that recombination rates are higher for female meioses than for males. For close relationships, this fact could be used to inform if DNA is more likely to be inherited via the paternal or maternal line. Further, we assumed Haldane’s model of recombinations, which assumes the absence of chiasma interference.

In future work, the simplifying modelling assumptions may be relaxed. There is also scope to investigate the use of IBD on the X chromosome. As far as we are aware, the evidential value of IBD segments on the X chromosome has not been quantified for distinguishing pedigree relationships. Finally, we mention that the current work only considered IBD for two pedigree members at a time. A natural generalisation would be to consider the problem of identifying the most likely relationship on the basis of IBD between more than two pedigree members. For example, given a pedigree with persons *a* and *b*, one could ask where person *c* would fit best in the pedigree given identified IBD segments between *c* and *a* and *c* and *b*.

## Figures and Tables

**Table 1:** Example of simulated IBD segments for a pair of first cousins on a chromosome of length 100 cM. **(a)** shows IBD segments only, while **(b)** shows the full IBD vector as both integer indices and their corresponding binary representations.

(a) Simulated IBD Segments Only			
Start (cM)	End (cM)	Length (cM)	IBD State ($X_{t}$)
0.00	5.06	5.06	1
5.06	29.06	24.00	0
29.06	93.94	64.88	1
93.94	100.00	6.06	0
**(b) Unobserved IBD vector represented by an integer and the binary representation**			
**Start (cM)**	**End (cM)**	**Length (cM)**	**IBD vector** **(** $V_{t}$ **)**
0.00	5.06	5.06	47: [0 0 1 0 1 1 1 1]
5.06	26.69	21.63	63: [0 0 1 1 1 1 1 1]
26.69	27.81	1.12	191: [1 0 1 1 1 1 1 1]
27.81	29.06	1.25	183: [1 0 1 1 0 1 1 1]
29.06	47.96	18.90	167: [1 0 1 0 0 1 1 1]
47.96	49.91	1.96	175: [1 0 1 0 1 1 1 1]
49.91	78.40	28.49	167: [1 0 1 0 0 1 1 1]
78.40	93.94	15.54	135: [1 0 0 0 0 1 1 1]
93.94	100.00	6.06	199: [1 1 0 0 0 1 1 1]

**Table 3:** Summary of 10,000 draws from LR distributions for distinguishing between linear relationships of order *d* and d+1.

$H_{1}$	$H_{2}$	$H_{\mathrm{true}}$	Pr(LR>1)	Pr(LR<1)	Median logLR	Accuracy
d=2	d=3	$H_{1}$	0.9837	0.0163	4.1709	0.983
		$H_{2}$	0.0177	0.9823	−3.5500	
d=3	d=4	$H_{1}$	0.9069	0.0931	1.7197	0.9123
		$H_{2}$	0.0824	0.9176	−1.4897	
d=4	d=5	$H_{1}$	0.8337	0.1663	0.9137	0.8397
		$H_{2}$	0.1544	0.8456	−0.8139	
d=5	d=6	$H_{1}$	0.7545	0.2455	0.4865	0.7743
		$H_{2}$	0.2060	0.7940	−0.4883	

**Table 4:** Summary of 10,000 draws from LR distributions for distinguishing between cousin relationships.

$H_{1}$	$H_{2}$	$H_{\mathrm{true}}$	Pr(LR>1)	Pr(LR<1)	Median logLR	Accuracy
1C	2C	$H_{1}$	0.9986	0.0014	8.5754	0.9990
		$H_{2}$	0.0007	0.9993	−7.6095	
2C	3C	$H_{1}$	0.9342	0.0658	2.4376	0.9421
		$H_{2}$	0.0501	0.9499	−1.8169	
3C	4C	$H_{1}$	0.7770	0.2230	0.7456	0.8119
		$H_{2}$	0.1532	0.8468	−0.6617	
4C	5C	$H_{1}$	0.7009	0.2991	0.1516	0.6980
		$H_{2}$	0.3049	0.6951	−0.3655	

**Table 5:** Numerical summaries of random samples from LR distributions ($H_{1}$ true and $H_{2}$ true) for distinguishing relationships of the same degree. For each value of $\kappa_{1}$ and each relationship, likelihood ratios comparing $H_{1}$ (the relationship) and $H_{2}$ (another relationship with the same $\kappa_{1}$) were sampled (1,000,000 of each).

$\kappa_{1}$	$H_{1}$	$H_{2}$	$H_{\mathrm{true}}$	Pr(LR>1)	Pr(LR<1)	Median logLR	Accuracy
1/2	GP	$\bar{\mathrm{GP}}$	$H_{1}$	0.9944	0.0056	4.7944	0.9946
			$H_{2}$	0.0052	0.9948	−8.1254	
1/2	HS	$\bar{\mathrm{HS}}$	$H_{1}$	0.9082	0.0918	1.0630	0.8823
			$H_{2}$	0.1436	0.8564	−1.7842	
1/2	N	$\bar{N}$	$H_{1}$	0.9023	0.0977	1.1379	0.8849
			$H_{2}$	0.1324	0.8676	−2.5758	
1/4	1C	$\bar{1C}$	$H_{1}$	0.8761	0.1239	0.8497	0.8509
			$H_{2}$	0.1742	0.8258	−1.1190	
1/4	GGP	$\bar{\mathrm{GGP}}$	$H_{1}$	0.9503	0.0497	1.7242	0.9385
			$H_{2}$	0.0734	0.9266	−2.9268	
1/4	GN	$\bar{\mathrm{GN}}$	$H_{1}$	0.8197	0.1803	0.3703	0.7465
			$H_{2}$	0.3267	0.6733	−0.3335	
1/4	HN	$\bar{\mathrm{HN}}$	$H_{1}$	0.8213	0.1787	0.4690	0.7637
			$H_{2}$	0.2938	0.7062	−0.4700	
1/8	1C1R	$\bar{1C1R}$	$H_{1}$	0.7938	0.2062	0.3913	0.7510
			$H_{2}$	0.2918	0.7082	−0.3773	
1/8	$G^{2}$GP	$\bar{G^{2}\mathrm{GP}}$	$H_{1}$	0.8637	0.1363	0.7782	0.8390
			$H_{2}$	0.1856	0.8144	−0.9586	
1/8	$G^{2}$N	$\bar{G^{2}N}$	$H_{1}$	0.7409	0.2591	0.1702	0.6578
			$H_{2}$	0.4253	0.5747	−0.0693	
1/8	H1C	$\bar{H1C}$	$H_{1}$	0.7375	0.2625	0.1660	0.6346
			$H_{2}$	0.4683	0.5317	−0.0322	

## Data Availability

The code to generate plots and tables is available from https://github.com/mkruijver/an_upper_bound_paper_data (accessed on 23 April 2025).
